# Possible Molecular Targets of Novel Ruthenium Complexes in Antiplatelet Therapy

**DOI:** 10.3390/ijms19061818

**Published:** 2018-06-20

**Authors:** Thanasekaran Jayakumar, Chia-Yuan Hsu, Themmila Khamrang, Chih-Hsuan Hsia, Chih-Wei Hsia, Manjunath Manubolu, Joen-Rong Sheu

**Affiliations:** 1 Graduate Institute of Medical Sciences, College of Medicine, Taipei Medical University, Taipei 110, Taiwan; tjaya_2002@yahoo.co.in (T.J.); gordanmilke1003@gmail.com (C.-Y.H); d119102013@tmu.edu.tw (C.-H.H.); d119106003@tmu.edu.tw (C.-W.H.); 2 Department of Life Sciences, National Chung Hsing University, Taichung 402, Taiwan; 3 Department of Chemistry, North Eastern Hill University, Shillong 793022, India; themmilakhamrang@gmail.com; 4 Department of Evolution, Ecology and Organismal Biology, Ohio State University, Columbus, OH 43212, USA; manubolu.1@osu.edu; 5 Department of Pharmacology, School of Medicine, College of Medicine, Taipei Medical University, Taipei 110, Taiwan

**Keywords:** ruthenium complex, antiplatelet, antithrombosis, signaling cascades

## Abstract

In oncotherapy, ruthenium (Ru) complexes are reflected as potential alternatives for platinum compounds and have been proved as encouraging anticancer drugs with high efficacy and low side effects. Cardiovascular diseases (CVDs) are mutually considered as the number one killer globally, and thrombosis is liable for the majority of CVD-related deaths. Platelets, an anuclear and small circulating blood cell, play key roles in hemostasis by inhibiting unnecessary blood loss of vascular damage by making blood clot. Platelet activation also plays a role in cancer metastasis and progression. Nevertheless, abnormal activation of platelets results in thrombosis under pathological settings such as the rupture of atherosclerotic plaques. Thrombosis diminishes the blood supply to the heart and brain resulting in heart attacks and strokes, respectively. While currently used anti-platelet drugs such as aspirin and clopidogrel demonstrate efficacy in many patients, they exert undesirable side effects. Therefore, the development of effective therapeutic strategies for the prevention and treatment of thrombotic diseases is a demanding priority. Recently, precious metal drugs have conquered the subject of metal-based drugs, and several investigators have motivated their attention on the synthesis of various ruthenium (Ru) complexes due to their prospective therapeutic values. Similarly, our recent studies established that novel ruthenium-based compounds suppressed platelet aggregation via inhibiting several signaling cascades. Our study also described the structure antiplatelet-activity relationship (SAR) of three newly synthesized ruthenium-based compounds. This review summarizes the antiplatelet activity of newly synthesized ruthenium-based compounds with their potential molecular mechanisms.

## 1. Introduction

Platelets in atherosclerotic process and consequently in the pathophysiology of cardiovascular disease is essential, as platelets, in addition to their contribution to thrombosis and hemostasis, modulate inflammatory reactions and immune response. Platelet activation is considered to be highly associated with cancer progression. The impact of platelets in cancer development has been suggested to be an organized process that causes the pathobiology of cancer growth. Platelets play a critical role in cancer metastasis, counting tumor cell migration, and invasion [1]. Platelet contents are released into the peritumoral space after platelet activation and enhance tumor cell extravasation and metastases [2]. A potential obstruction that surrounds chronic administration of antiplatelet agents in the setting of active malignancy is directly related to the principal role that platelets play in maintaining hemostasis. An in vivo reduction of pulmonary metastases was found in a murine model of breast cancer by the platelet aggregation inhibitor cilostazol [3]. Wenzel et al. observed decreased ex vivo platelet aggregability and reduced platelet-tumor complex formation while administrating liposomal cilostazol [3]. The current antiplatelet agents permanently inhibit their target in inhibiting platelet aggregation; however, the bleeding risk is still difficult to mitigate. Therefore, the progress of harmless and potential therapeutic strategies for the prevention and treatment of thrombotic diseases is still required.

## 2. Ruthenium Metal Complexes

**Figure 1:**
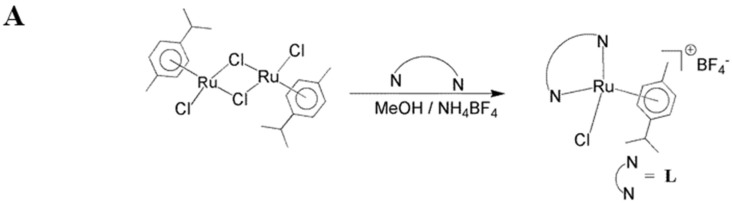

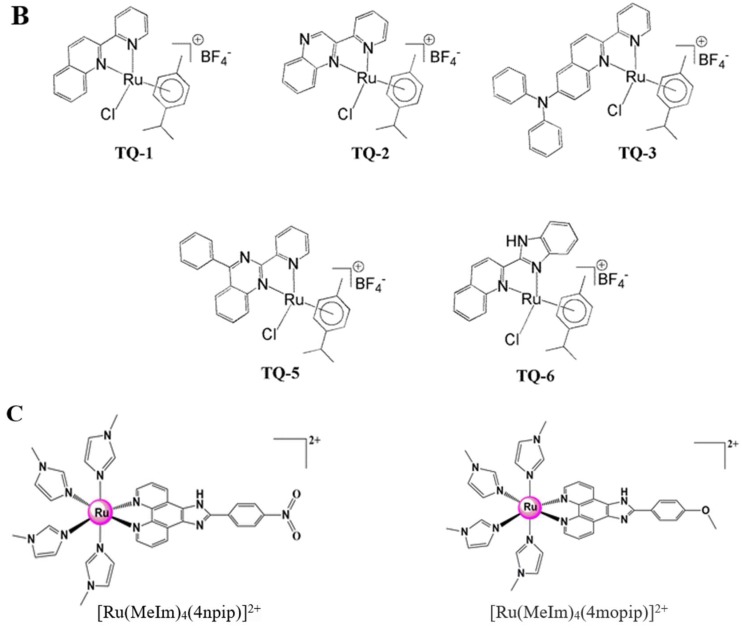
Synthetic procedure of the ligands (**A**) and its complexes (**B**) of TQ-1, TQ-2, TQ-3, TQ-5 and TQ-6; (**C**) Structures of ruthenium (II) methylimidazole complexes [Ru(MeIm)_4_(4npip)]^2+^ and [Ru(MeIm)_4_(4mopip)]^2+^.

Inorganic medicinal chemistry is a rich area for controlling several diseases via the development of new therapeutic agents based on bioactive metal complexes. Cisplatin has been widely used as an antimetastatic drug for treating ovarian and testicular cancers for a long time [4]. The platinum diammolino compounds, cisplatin and carboplatin, have limitation due to their dose-limiting side effects and resistance after repeated use in treatment [5]. To solve these restrictions, the screening for anticancer activity among complexes of other metals has received much attention. Currently, ruthenium complexes are found to be striking alternatives for platinum because of several favorable properties suited to rational anticancer drug design and biological applications [6]. Therefore, ruthenium metal complexes are considered the most encouraging anticancer agents. Until now, two ruthenium complexes, NAMI-A and KP1019 have entered clinical trials. NAMI-A is effective against lung metastases [7]. In recent years, abundant progress has been made in the anticancer activity of ruthenium (II) polypyridyl complexes, as many Ru(II) polypyridyl complexes, show motivating anticancer activity [8,9]. A previous study shows [Ru(phen)_2_(biim)](ClO_4_)_2_ inhibits the growth of HeLa cells via stimulating the apoptotic cell death [10]. Ruthenium polypyridyl complex [Ru(phen)_2_(dbtcp)]^2+^ found high mitochondria specificity, greater photostability, high resistance to the loss of mitochondrial membrane potential and considerable tolerance to environmental change [11]. To gather more insight into the bioactivity of Ru (II) complexes toward cancer cells, these compounds use various medical applications principally due efficient biological activity against some types of diseases. Recent studies found that ruthenium complexes possess potential antiplatelet and antithrombotic effects [12,13,14]. Some of the novel ruthenium complexes (TQ-1, TQ-2, TQ-3, TQ-5 and TQ-6) were prepared by following the reported methods [15]. Illustrative synthetic procedures of the ligands and complexes is shown in Figure 1A,B. The structures of ruthenium (II) methylimidazole complexes [Ru(MeIm)_4_(4npip)]^2+^ and [Ru(MeIm)_4_(4mopip)]^2+^ were given in Figure 1C. The molecular structures of some of the complexes were investigated by single-crystal X-ray studies.

## 3. Antiplatelet Effects of Ruthenium Compounds

**Figure 2:**
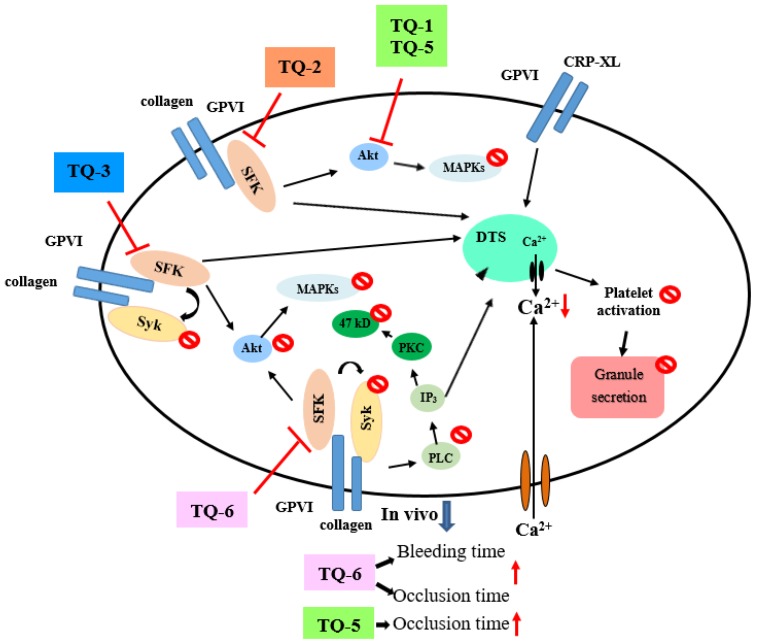
General representation of the main targets and proposed mechanisms of action of ruthenium compounds as antiplatelet drugs. 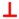 inhibits; 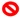 blocks; 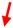 decrease; 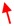 increase.

Various chemical and biological properties displayed by ruthenium complexes make these molecules very interesting for developing new drugs. In vitro and in vivo studies established that numerous ruthenium-based compounds show high cytotoxicity towards a wide range of cancer cells with reduced side effects [16,17]. Fortunately, ruthenium-based complexes are not affected by platinum-induced resistance mechanisms. Although there are several in vitro and in vivo biological studies that have shown that ruthenium-based compounds have potent anticancer activity with condensed side effects, to date, no study exists that has investigated the effects of ruthenium compounds on platelet aggregation. Our recent study established that a novel ruthenium-based compound TQ-5 suppressed platelet aggregation in vitro in washed human platelets by inhibiting the phosphorylation of Akt and JNK1, and subsequently reducing the ATP release reaction and intracellular calcium mobilization [12]. Another interesting study from our group also showed ruthenium compound TQ-6 has a novel role in inhibiting platelet activation through the inhibition of the agonist receptor-mediated inside-out signaling such as Src-Syk-PLCγ2 cascade and subsequent suppression of granule secretion, leading to disturbance of integrin α_IIb_β_3_-mediated outside-in signaling, and ultimately inhibiting platelet aggregation [13]. Our recent SAR study also showed that among the three newly synthesized ruthenium-based compounds, TQ-3 has potently inhibited platelet aggregation in vitro, and this inhibitory effect was attributed to the suppression of Syk-Lyn-Fyn cascade and subsequent destruction of Akt, JNK and p38MAPKs activation [18]. Moreover, this study also found TQ-3 reduces the level of ATP, surface P-selectin expression and [Ca^2+^]_i_ and ultimately inhibits platelet aggregation. The incorporation of the additional nitrogen atom on the quinoline ring of TQ-1 ligand to obtain TQ-2 and TQ-5 respectively causing the change in electronic nature of ligand that enhances the electron density on the aromatic rings. Variation of ligands pK_a_ values tunes the aquation rate of the chloride ion by water molecule. Pyridine, 5.2 and Quinoline, 4.9 (TQ-1 and TQ-3); Quinoxaline, 0.60 (TQ-2); Benzimidazole, 5.3 (TQ-6); Quinazoline, 3.51 (TQ-5). TQ-3: The coordinated ligand containing freely rotating N-diphenyl amine group feasibly restricts the strong interaction between the complex and the biological systems. TQ-5: DNA binding affinity may be enhanced by the substitution of phenyl ring on the quinazoline core and improves the hydrophobic interaction and planarity of the molecule. TQ-6: NH functionality on the ligand modulates the lipophilicity/hydrophilicity. These characteristic features might be credited to the observed antiplatelet effects of TQ-3, TQ-5 and TQ-6 (Figure 2). This hypothesis can be further justified from the studies conducted by Giannini et al. [19] and Gorle et al. [20]. These authors proposed that the in vitro activity of anticancer drugs can regularly be associated in part, to their lipophilic character; higher hydrophobicity may contribute to an increased uptake of the complex by the cells, thereby enhancing the anticancer activity. These studies establish the importance of ruthenium-based organometallic complexes in the development of novel anti-platelet agents for the prevention and treatment of thrombotic diseases.

### 3.1. Ruthenium Compounds on ATP and [Ca^2+^]_i_ Mobilization in Antiplatelet Therapy

Secretion of platelets is an agonist-induced response, which is of importance for the enhancement of platelet activation and for the various effects of secreted platelet constituents in other tissues. The releasable substances are stored in dense granules, the a-granules and acid hydrolase-containing granules of platelets. Upon stimulation, the contents of these granules are rapidly emptied into the surrounding media by exocytosis. Human platelets are responsible for an ATP-dependent proteolysis of several regulatory proteins for cellular processes [21]. Protein degradation by 20S proteasomes in vivo requires ATP hydrolysis and the homologous ATPases in the eukaryotic 26S proteasome [22]. Therefore, the inhibition of 20S proteasome in platelets does not influence the collagen, epinephrine and thrombin dependent aggregating mechanisms. Interestingly, a previous study found that bortezomib, a first proteasome inhibitor entered to the clinical trials, holds antiproliferative and proapoptotic effects in patients with multiple myeloma [23]. These authors suggested that the anti-aggregating effects of bortezomib may be related to adenine nucleotide receptor dependent regulatory proteins which are essential for physiological and pathophysiological cellular processes.

Multiple studies revealed that ATP could stimulate its own release via P2Rs both in an autocrine and paracrine manner. This mechanism permits regenerative signal amplification via positive feedback and ATP-mediated propagation of the ATP-induced signal. Cell-to-cell spread of Ca^2+^ signals mediated through ATP receptors had observed early in rat basophilic leukemia cells and mast cells [24]. The authors suggested that extracellular ATP accelerates the release of secretory granules containing additional ATP by triggering intracellular Ca^2+^ in quiescent neighboring cells or in cells, which have begun to degranulate, thus amplifying the initial response. Another study showed that addition of ADP and a variety of nucleotide analogs stimulated ATP release through P2Y receptors from endothelial cells isolated from guinea pig heart [25]. Correspondingly, ATP induced the release of ATP in cultured human umbilical vein endothelial cells resulting in maintained extracellular ATP concentrations. This implicated a self-perpetuating mechanism of ATP-induced ATP release likely to play a role in local vascular control [26].

**Figure 3:**
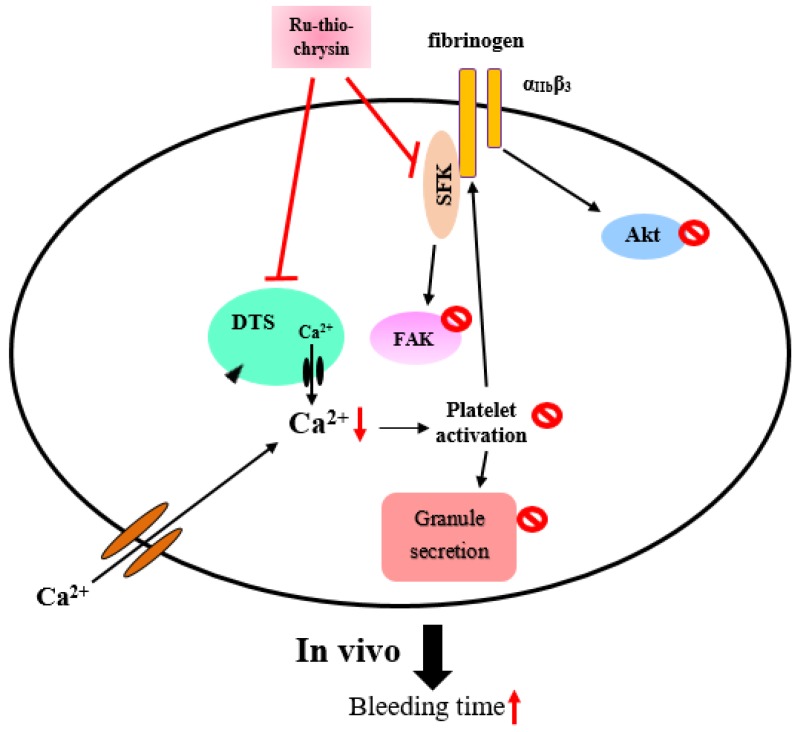
Molecular targets of Ru-thio-chrysin to its inhibitory effects on platelet function.

Thrombin, collagen, and ADP commonly generate numerous aggregation-inducing molecules, such as Ca^2+^, thromboxane (TxA_2_), etc. TxA_2_ produces IP_3_ to mobilize [Ca^2+^]_i_ through the G-protein-coupled receptor/PLC-β pathway, and constricts the blood vessel tract [27], which enforces thrombus formation. A lot of agonists such as collagen, thrombin, and ADP mobilize [Ca^2+^]_i_ to phosphorylate Ca^2+^/calmodulin-dependent myosin light chain, which plays a role in secretion of granules such as serotonin and ATP [28], and platelet aggregation. Therefore, the inhibition of [Ca^2+^]_i_ mobilization and ATP production are very important for evaluating the antiplatelet effect of a substance. Our recent studies demonstrated that novel ruthenium complexes TQ-5 and TQ-6 potently inhibited collagen-induced [Ca^2+^]_i_ mobilization and ATP production in human platelets, representing that TQ-5 and TQ-6 inhibits platelet aggregation through suppressing [Ca^2+^]_i_ mobilization and ATP production (Figure 2). Another structural antiplatelet-activity relationship study of newly synthesized ruthenium (II) complexes, TQ-1, TQ-2 and TQ-3 in agonists-induced washed human platelets revealed that TQ-3 compound was effective at inhibition of collagen-induced ATP release, calcium mobilization ([Ca^2+^]_i_) without cytotoxicity [18]. A recent study from Ravisankar et al. [14] measured ATP in washed platelets activated by cross-linked collagen-related peptide (CRP-XL) in the presence and absence of different concentrations of chrysin and Ru-thio-chrysin. They found both chrysin and Ru-thio-chrysin significantly inhibited ATP (dense granule secretion). In addition, they found Ru-thio-chrysin exhibited significantly greater effects on inhibiting Ca^2+^ in platelet rich plasma compared to chrysin alone (Figure 3). These data demonstrate that ruthenium and its derivatives affect platelet granule secretion and calcium mobilization, which may influence the subsequent functions of platelets.

### 3.2. Ruthenium Compounds on MAPKs in Antiplatelet Effects

Mitogen-activated protein kinases (MAPKs) are serine/threonine kinases that regulate cellular proliferation and stimulators such as growth factors and hormones. MAPKs consist of four subgroups: p38, extracellular stimuli-responsive kinase (ERK), and c-Jun NH2-terminal kinase (JNK) and big mitogen-activated protein kinase 1 (BMK1; ERK5). Of these, ERK, JNK and p38 have been identified in platelets and regulated by an extensive range of receptors. In response to the platelet agonists, both p38 and ERK are activated, and the highest activity is demonstrable within minutes of agonist activation [29]. The agonist-induced activation of p38 and ERK seems temporary, possibly due to its negative regulation by integrin outside-in signaling. Ligand binding to integrin α_IIb_β_3_ was found to down-regulate active p38 and ERK in platelets, ERK2 phosphorylation appears to regulate MEK 1/2 and PKC [30], and JNK was also recognized in platelets in a similar way to ERK2. The characters of JNK and ERK2 in physiopathology are uncertain and had been proposed as suppressors of α_IIb_β_3_ activation or negative regulators of platelet activation [31]. Additionally, p38 provides a crucial signal that is necessary for aggregation induced by collagen or thrombin [32]. Among the numerous downstream targets of p38, cPLA2 is the most physiologically relevant in platelets, which catalyzes arachidonic acid (AA) release to produce TxA2 [33]. Therefore, p38 MAPK appears to provide a TxA2-dependent platelet aggregation pathway. Stimulation of platelets with various agonists resulted in Akt activation. It is known that Akt plays a role as one of the numerous downstream effectors of PI-3 kinase [34].

Studies have established that ERK2, JNK1, and p38MAPK play essential roles in ruthenium complex-mediated inhibition of platelet activation. In one study, the authors found that novel ruthenium compound TQ-5 dose-dependently suppressed collagen-induced Akt and JNK phosphorylation, whereas it does not affect the p38MAPK and ERK phosphorylation [12]. In that study, TQ-5 significantly demolished phosphorylation of Akt and JNK at a maximum concentration of 5 µM (Figure 2). These findings settled that Akt/JNK signaling implicate TQ-5’s antiplatelet activity [12]. Another newly synthesized ruthenium compound TQ-6 was notably inhibited the phosphorylation of ERK2, JNK1, and p38 MAPK, representing that the inhibition of the MAPKs signaling pathways is crucial to the TQ-6-mediated inhibition of platelet activation [13]. A recent study was found where among the three novel synthesized ruthenium compounds TQ-1, TQ-2 and TQ-3, only TQ-3 inhibited Akt, JNK and p38 phosphorylation induced by collagen [18].

### 3.3. Ruthenium Compounds on Cyclic AMP and Cyclic GMP Signaling in Platelets

Cyclic nucleotides (cAMP- and cGMP) have long been accepted as potent inhibitors of platelet aggregation. Human platelet activation inhibited via intracellular cAMP- and cGMP-mediated pathways, and the importance of cyclic nucleotides in controlling platelet activation was confidently proven [35]. Cyclic GMP have been observed to inhibit platelet functions for several decades [36]. Abnormal cyclic nucleotide signaling in platelets might play a role in common diseases such as ischemic heart disease, heart failure, and diabetes [37]. Defects in prostacyclin signaling reduce cAMP levels, resulting in hyperactive platelets and a pro-thrombotic state [38]. cAMP and/or cGMP-elevating agents have shown clinical benefit as platelet inhibitors. For instance, dipyridamole in combination with low-dose aspirin is an approved therapy for stroke prevention [39]. Dipyridamole elevates cAMP levels in platelets by several mechanisms, including inhibition of phosphodiesterase (PDE)-mediated breakdown. Groups of compounds that activate cGMP production by soluble guanylyl cyclase have been shown to reduce thrombus formation [40]. cAMP and cGMP can block many aspects of platelet activation, including early activator signals such as release of Ca^2+^ from intracellular stores and G-protein activation, and adhesion, granule release, aggregation, and apoptosis [41]. Therefore, the adhesion, activation, and aggregation of platelets is a many stepped progression and pharmacological targeting of platelet activating factors and their receptors has become a main strategy in antithrombotic drug development.

Antiplatelets may inhibit platelet aggregation via increasing either cGMP or cAMP; therefore, these nucleotides can be measured in platelets stimulated in the presence of 9-(tetrahydro-2-furanyl)-9*H*-purin-6-amine (SQ22536), an adenylyl cyclase inhibitor and 1*H*-[1,2,4]oxadiazolo[4,3-a]quinoxalin-1-one (ODQ), a soluble guanylyl cyclase inhibitor. Our studies observed that neither SQ22536 nor ODQ significantly reversed the TQ-5 and TQ-6-mediated inhibition of collagen-induced platelet aggregation. Therefore, ruthenium complexes TQ-5 and TQ-6-mediated inhibition of platelet activation is independent of intracellular cyclic nucleotide formation [12,13].

### 3.4. Molecular Targets of Ruthenium Compounds in Antiplatelets Property

Different molecules have serious roles in defining cellular activity with distinct structures and function [42]. An understanding of how ruthenium complexes interact with specific targets within cells is therefore important for exploring the antiplatelet mechanism and choosing the most potent ruthenium complex for selective and effective therapy. In recent years, studies have been devoted to elucidating the signaling events downstream of GPVI and has described the role of signaling molecules that negatively regulate Syk activity [43,44]. A study explored the negative regulation of Syk by PKC in GPVI signaling. In human platelets, inhibition of PKC leads to Syk hyperphosphorylation on residues Tyr-525/526 whereas Tyr-323 and Tyr-352 phosphorylations are unaffected [45]. These authors have also found PKC negatively regulates Syk activity, since it induces hyperphosphorylation of downstream targets, PLCγ2 upon PKC inhibition. PLC activation results in IP_3_ and DAG production, which triggers PKC, thus tempting p47 phosphorylation [46]. PLCs are characterized into six families, PLCβ, PLCγ, PLCδ, PLCε, PLCζ, and PLCη [47], and contain the isozymes of PLCγ1 and PLCγ2. PLCγ2 plays a role in collagen-dependent signaling in platelets [48].

Src family kinases (SFKs) contributed towards the regulation of several cellular events such as proliferation, differentiation, motility, and adhesion [49]. Studies have suggested that there are nine members of the Src family that include Src, Lck, Hck, Blk, Fyn, Lyn, Yes, Fgr, and Yrk [50,51]. Earlier studies designated that both human and rodent platelets contain high levels of Src as well as Fyn, Lyn, Hck, Yes, Lck, and Fgr [52]. Although platelets contain high levels of SFKs, their role in platelet function is not clarified yet. SFKs, principally Lyn and Fyn, play essential roles downstream of collagen receptors in platelets [53]. Studies have suggested that platelets from Fyn^−/−^ mice exhibited delayed spreading on immobilized fibrinogen coated surfaces [54], whereas another study found platelets from Lyn^−/−^ mice poorly spreading on von Willebrand factor [55]. Studies have also proven that SFKs play a role in thromboxane generation, shape change, as well as regulation of phosphorylation of Akt [56] and ERK [57]. Our recent studies established that ruthenium compound TQ-6 evidently diminished collagen-induced PLCγ2-PKC activation (Figure 2) but had no direct effects on PKC activation because it did not inhibit PDBu-induced platelet aggregation [13]. This result indicated that TQ-6-mediated inhibition of platelet activation involves PLCγ2 downstream signaling and clarifies how TQ-6 is more effective in inhibiting collagen-induced platelet aggregation than that induced by thrombin. Another study found among the ruthenium complex, TQ-3 perceptibly reduced collagen-induced phosphorylation of Fyn, Lyn and Syk; however, TQ-1 and TQ-2 had no direct effects on these proteins, suggesting that TQ-3-mediated inhibition of platelet activation involves SFKs and Syk downstream signaling. Ru-thio-chrysin was found to inhibit Akt and FAK phosphorylation induced by CRP-XL (Figure 3), and this inhibitory effect may directly or indirectly influence other signaling pathways that render the inhibition of platelet function [14]. Ru-thio-chrysin was also found to have a significant impact on the dephosphorylation of Src at Y527, which is an essential phenomenon for the activation of platelets [14].

## 4. Antithrombotic Effect of Ruthenium Compounds

Platelets are vital to the progress of the pathological thrombus liable for cardiovascular disease [58]. Thrombosis can be described as the formation of a blood clot (thrombus) within a blood vessel [59]. This process is considered pathologic except in the case of traumatic injury, where thrombus formation may stop blood loss and protect against systemic infection. Aspirin, the most popular drug in the world, reduces platelet abnormal function by irreversibly inhibiting platelet cyclooxygenase (COX-1), responsible for the ultimate production of thromboxane A2, an activator of the coagulation cascade [60]. Although aspirin is effective on COX-1, it also affects COX-2. Aspirin therapy increases bleeding risk, particularly gastrointestinal. Small bowel bleeding with low dose aspirin therapy was found to be more common in patients. Clopidogrel permanently inhibits the P2Y12 purinergic receptor on platelets, preventing stimulation by adenosine diphosphate, thus inhibiting platelet aggregation [61].

In a previous study for thrombosis, the mesenteric venules were continuously irradiated by fluorescein sodium, resulting in considerable damage to the endothelial cells [62]. Despite aspirin being the most effective antiplatelet drug for preventing or treating cardiovascular diseases, it prolongs the bleeding time. For instance, in the tail transection model of mice, after 30 min of dosing 150 mg/kg aspirin through an intraperitoneal rout, the bleeding time was found to be considerably increased from 229.2 ± 20.5 s to 438.1 ± 22.6 s. In our recent ex vivo study, a shear-induced platelet plug formation in whole blood was analyzed [13]. The PFA-100 system was used to mimic the in vivo conditions of blood vessel injury, in which platelets were exposed to a high shear rate to record the time required for platelet aggregation to occlude an aperture in a collagen-coated membrane. The closure time (CT) of the collagen/epinephrine (CEPI)-coated membrane in whole blood treated with the solvent control (0.5% DMSO) was 93.2 ± 5.5 s. However, treatment with 1 μM TQ-6 significantly increased the CT of the CEPI-coated membranes (123.8 ± 5.7 s), indicating that the adherence of platelets to collagen was prolonged under flow conditions after 1 μM TQ-6 treatment. Similarly, the bleeding time in 0.4 mg/kg of the ruthenium compound TQ-6-treated mice was slightly longer than that of the solvent control (0.5% DMSO)-treated mice (Figure 2). Ru-thio-chrysin was reported to extend the bleeding time in mice (Figure 3). These results indicating that the prolongation of the bleeding time may at least partly be induced by the antiplatelet activity of TQ-6 and Ru-thio-chrysin.

## 5. Safety and Toxicity of Ruthenium Compounds in Platelets

The uptake of ruthenium complexes by cells is essential for effective antiplatelet therapy. To move into living cells, molecules and atoms must cross or enter the cell membrane. The cell membrane comprises different proteins and lipids, and its function is to control what substances enter the cells for the beneficial or toxic effects.

Previous studies had examined the toxic effects of ruthenium compounds in platelets by measuring extracellular activity of lactate dehydrogenase (LDH). In the most eukaryotic cells, LDH is present and released into the culture medium upon cell death due to the damage of plasma membrane. The LDH study revealed that ruthenium compound TQ-1 and TQ-2 (up to 250 μM), TQ-3 (5 μM), TQ-5 (3–10 μM) and TQ-6 (20–200 μM) incubated with platelets for 20 min did not significantly increase LDH activity in platelets [12,13,18], indicating that ruthenium compounds are safe and they do not affect platelet permeability or induce platelet cytolysis. The in vitro cytotoxic effects of ruthenium (II) methylimidazole complexes [Ru(MeIm)_4_(4npip)]^2+^ and [Ru(MeIm)_4_(4mopip)]^2+^ against four different human cancer cell lines (A549, NCI-H460, MCF-7, HepG2) and one normal cell line (HBE) was analyzed by 3-(4,5-dimethylthiazol-2-yl)-2,5-diphenyltetrazolium bromide (MTT) assay [63]. This study found that both complexes exhibit higher cytotoxicity in four cancer cell lines than in normal cells, suggesting that these complexes have high selectivity between tumor cells and normal cells [63]. Another important in vivo study established that the highest intraperitoneal dose (10 mg/kg) of ruthenium-II complex, *cis*-(Ru[phen]_2_[ImH]_2_)^2+^ significantly reduced tumor volume and weight, induced oxidative stress in tumor tissue, reduced the respiration of tumor cells, and induced necrosis without inducing apoptosis in the tumor [64]. More importantly, there was no clinical signs of toxicity or death in tumor-bearing or healthy rats that were treated with *cis*-(Ru[phen]_2_[ImH]_2_)^2+^ [64]. These results suggested that *cis*-(Ru[phen]_2_[ImH]_2_)^2+^ has antitumor activity through the modulation of oxidative stress and impairment of oxidative phosphorylation, thus promoting cancer cell death without causing systemic toxicity. 

Ruthenium complexes Δ-[Ru(bpy)_2_(HPIP)](ClO_4_)_2_ bpy = 2,2′-bipyridine and HPIP = 2-(2-hydroxyphenyl)imidazo[4,5-f][1,10]phenanthroline presented high affinity for cancer cells in vitro [65], with no identified side effects on kidney, liver, peripheral nervous system, or the hematologic system, at the pharmacologically effective dose in vivo [66].

## 6. Conclusions

Despite current antiplatelet therapy preventing death and disability in patients with high risk of thrombotic disease by inhibiting thrombotic events, the lack of efficiency and inconsistent clinical procedures remains a problem. Therefore, a better understanding of platelet function is essential for discovering new antiplatelet approaches with improved clinical outcomes. Ruthenium compounds display fascinating anticancer activity in in vitro and in vivo models. Compared to platinum compounds, ruthenium is coordinated at two additional axial sites and it tends to form octahedral compounds. Commonly, the ligand arrangement and organization geometry between ruthenium and its ligands primarily regulate the activity of ruthenium compounds, especially to their reactivity, hydrophobicity, binding, cellular uptake and intracellular distribution. From this perspective, data presented in this review demonstrates that different classes of ruthenium compounds possess significant antiplatelet effects via multiple targets (Figure 2 and Figure 3). The information of both platelet biology and the functions of ruthenium compounds used for antiplatelet therapy will provide new opportunities to develop therapeutic strategies aimed at promoting cerebro/cardiovascular health.
